# Cellular Senescence, Inflammation, and Cancer in the Gastrointestinal Tract

**DOI:** 10.3390/ijms24129810

**Published:** 2023-06-06

**Authors:** Egan L. Choi, Negar Taheri, Abhishek Chandra, Yujiro Hayashi

**Affiliations:** 1 Graduate Research Education Program (Choi), Mayo Clinic, Rochester, MN 55905, USA; choi.egan@mayo.edu; 2 Department of Physiology and Biomedical Engineering (Taheri, Chandra and Hayashi), Mayo Clinic, Rochester, MN 55905, USA; taheri.negar@mayo.edu (N.T.); chandra.abhishek1@mayo.edu (A.C.); 3 Division of Gastroenterology and Hepatology (Taheri and Hayashi), Mayo Clinic, Rochester, MN 55905, USA; 4 Robert and Arlene Kogod Center on Aging (Chandra), Mayo Clinic, Rochester, MN 55905, USA

**Keywords:** senescence, inflammation, cancer, aging, gastrointestinal tract

## Abstract

Due to modern medical advancements, greater proportions of the population will continue to age with longer life spans. Increased life span, however, does not always correlate with improved health span, and may result in an increase in aging-related diseases and disorders. These diseases are often attributed to cellular senescence, in which cells become disengaged from the cell cycle and inert to cell death. These cells are characterized by a proinflammatory secretome. The proinflammatory senescence-associated secretory phenotype, although part of a natural function intended to prevent further DNA damage, creates a microenvironment suited to tumor progression. This microenvironment is most evident in the gastrointestinal tract (GI), where a combination of bacterial infections, senescent cells, and inflammatory proteins can lead to oncogenesis. Thus, it is important to find potential senescence biomarkers as targets of novel therapies for GI diseases and disorders including cancers. However, finding therapeutic targets in the GI microenvironment to reduce the risk of GI tumor onset may also be of value. This review summarizes the effects of cellular senescence on GI aging, inflammation, and cancers, and aims to improve our understanding of these processes with a goal of enhancing future therapy.

## 1. Introduction

According to the World Health Organization, 1.4 billion people globally will be older than 60 years in 2030 [1]. As a result, a considerable proportion of the population will be subject to aging-related health conditions, such as problems related to cellular senescence, a key driver of aging and aging-associated diseases, and also a higher risk of cancer. The incidence of gastrointestinal tract (GI) diseases, including cancers increases with age, partially due to the accumulation of senescent cells [2], and will no doubt increase further as the older population expands within the current decade. One therapeutic option in GI cancers may be to regulate cellular senescence, which is defined as a state of irreversible cell cycle arrest that typically occurs in response to DNA damage and the acquisition of a proinflammatory secretory phenotype. Although the aberrant accumulation of senescent cells is associated with aging-related diseases/disorders, this state can also be used to manage tumors. For instance, given the risk of toxicity from extreme drug dosages in chemotherapies, the combination of pro-senescence and senolytic therapy may reduce that risk while amplifying cancer destruction [3]. The detailed roles of cellular senescence in GI cancers are not clear, but evidence indicates that the senescent fibroblast microenvironment can increase the risk of colon cancers [4]. The unclear relationship between cellular senescence and GI cancers serves as a motivation to further develop research efforts on this topic to expand therapeutic options for the future aging population of the world. 

Fragility in older persons often stems from multiple sources of disease, cascading effects caused by those sources, and accumulation of senescent cells. Older people are often at a higher risk for experiencing complications of several infectious diseases, such as COVID-19, intestinal bacterial overgrowth, and GI ulcers [5,6]. Many of these diseases can be attributed to senescence in some capacity; for example, COVID-19 overloads older persons with senescent cells, DNA damage accumulation, and inflammatory cytokines [7,8]. This could be detrimental in the long term, as it may lead to the development of other diseases and disorders if left untreated. Older persons are already affected by a greater morbidity from common GI disorders, such as esophageal reflux, dysphagia, chronic constipation, and gastroparesis (defined as delayed gastric emptying without mechanical blockage) [9,10]. Owing to the compounding effects of the high risk of infectious diseases, GI bleeding, and cancer progression, death is therefore more likely in the aging population. Thus, it is necessary to elucidate how the components and mechanisms of senescence interact to better understand how they contribute to disease development. 

The innate immunity, which acts as the first line of defense against invading pathogens including *Helicobacter pylori* (a Gram-negative spiral-shaped bacterium in human stomachs), is also a key player in aging and aging-associated diseases. Age-associated innate immune dysfunction can also predispose the elderly to fragility and more severe disease. We also discuss the implications of age-associated changes in innate immunity to better understand the biological basis of the age-associated chronic inflammatory GI diseases and cancers.

## 2. Cellular Senescence

**Figure 1:**
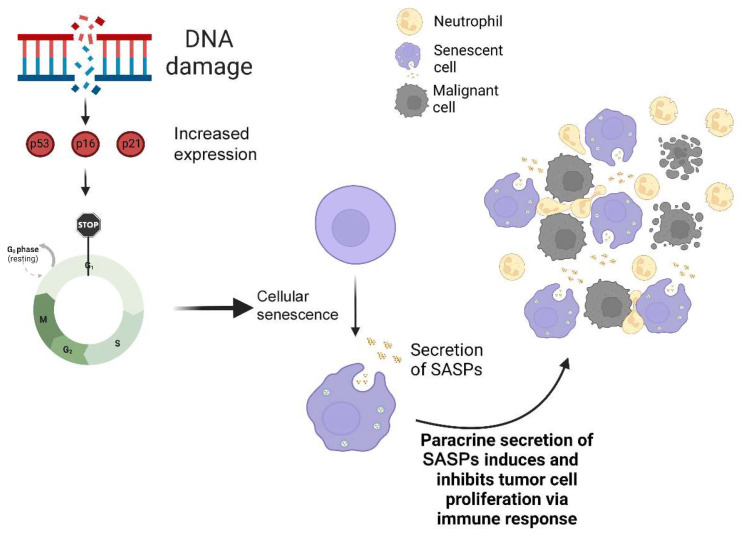
Cellular senescence and cancer development. Cellular senescence is a stress response pathway that elicits an irreversible and permanent cell cycle arrest, and releases inflammatory cytokines termed senescence-associated secretory phenotype proteins (SASPs), which typically recruit immune cells, such as neutrophils, to respond to malignant tumor cells.

Aging can be defined as the development of the human body from conception to death. Throughout this process, humans undergo an eventual decline of cellular function [11]. This decline is often associated with the accumulation of DNA damage and related cellular fates such as senescent cells, which results in the aging of the human body. Cellular senescence causes cells to undergo cell cycle arrest, at which point the cells are no longer able to grow and replicate but remain metabolically active so as to affect normal cells both locally and systemically [12]. It is an important defense mechanism against DNA damage, telomere destruction, and other stressors. Without senescence, DNA damage would rampantly increase tumor proliferation and progression [13]. The senescence pathway involves DNA damage, which activates tumor antigens such as the tumor suppressor p53 and cyclin-dependent kinase inhibitors p16 and p21, which then leads to cell cycle arrest and the increased generation of myriad cytokines, chemokines, and other inflammatory proteins from senescent cells (Figure 1). This protein expression pattern is termed the senescence-associated secretory phenotype (SASP). Telomere shortening or telomeric DNA damage can also lead to the activation of p16 and p21 [14], and stimuli such as cytokines or oncogene activity can also lead to cell cycle arrest and subsequent senescent phenotypic expression [15].

DNA damage is one of the main contributors to aging; it occurs naturally from metabolic processes such as the creation of oxidative byproducts by mitochondria [16]. Thus, DNA damage coincides with p53 expression, which is caused by mitochondrial apoptosis [17]; mitochondrial dysfunction, which increases p53 expression in senescent cells [18]; and p53 expression, which inhibits mitochondrial elongation in cell senescence [19]. Mitochondrial elongation is a mechanism that increases cell viability, which implies that p53 inherently increases the likelihood of cell apoptosis [20]. This is interesting, considering that senescence acts opposite to apoptosis, neither of which is directly correlated with the other. However, a recent study defined two downstream proteins of p53—PUMA, a key protein linked to p53-mediated apoptosis, and p21 (one of the genes linked to cellular senescence)as being significant contributors to lymphoma prevention [21]. p53 is involved in almost all DNA repair pathways. This implies that all three processes (cellular senescence, DNA repair, and apoptosis) work together through p53, which acts as a liaison. 

Hypothetically, these three processes may be dependent on the needs of the cell, as senescence or apoptosis may be preferred in different situations. This is possible given how different organs vary in response to aging, such as the intestinal cells being more likely to undergo apoptosis and implies that different epigenomic alterations to the genome occur in different cell types [22]. Therefore, various senescence studies are needed, especially in GI research, as the many different cells throughout the GI most likely lead to a great variety in senescence-associated gene expression. p53 has been implied to exhibit an epigenetic effect on cells as shown by its positive relationship with α-ketoglutarate, an intermediate of the citric acid cycle and a key substrate for several epigenetic processes [23]. Additionally, a recent in vitro study showed no effect of α-ketoglutarate on p21 expression in senescent cells but did show an effect on SASP-related cytokine expression [24]. This suggests that p53 may have an epigenetic effect on its own downstream SASP expression.

As shown in Figure 1, several SASP proteins, such as interleukin (IL)-8, are involved in the recruitment of immune cells, such as neutrophils, to impede malignant cell growth [25,26]. The main functions of SASP proteins are to promote cell proliferation, tissue repair, and stem cell renewal; all of these suggest that the goal of SASP is to repair damage and prevent cancer development [27,28]. 

SASP protein overexpression is known to cause tumor progression by creating an inflammatory environment that leads to epithelial–mesenchymal transitions, which then increases cancer cell invasion [29]. SASP proteins such as IL-6 and IL-8 have also been shown to interact with chemokines and oncogenic proteins to promote cancer growth [30,31]. As a result, there is value in accounting for SASP when researching oncogenic development in older persons [32]. Given that mutations are more likely to occur through aging, a mutation in p53 may lead to the dysregulation of SASP and to subsequent cancer development, which was shown in a study in zebrafish in which a p53 mutation supported such a phenomenon [33]. Such mutations are common in human tumors. Most oncogenic development may stem not from SASP protein expression caused by natural senescent cell build up, but rather mutations in the regulation of senescence.

## 3. Senescence in GI Mucosa and Muscle

The susceptibility of GI cells to the senescence process varies depending on the specific cell type and region of GI; the potential contribution of senescent GI cells to age-related changes in GI health has not been fully investigated [10]. Although cellular senescence seems to be prevalent in GI mucosa [34,35], interstitial cells of Cajal (ICCs; pacemaker and neuromodulator cells of GI motor function) and their progenitor cells are reduced in number and function through cell cycle arrest due to reduced ERK signaling rather than the senescence pathway [34]. Cellular senescence is not only cell dependent or organ dependent, but also may be region dependent, as different regions of the colon have shown marked differences in p16 expression [36]. Additionally, no senescence markers specific to the GI have been found [37,38], so this merits investigation, and will be critical for improving our understanding of the roles of senescent cells in the GI. It would be rash to apply currently used senescence markers from other organs to the GI.

## 4. Immunosenescence

Immunosenescence is the natural decline in the immune system’s function, which drives accelerated aging. Specifically, as humans age, senescence occurs increasingly in certain subsets of immune cells, such as T cells. In mice that were genetically modified to have an accelerated senescence of the immune system, T cell numbers were found to have significantly reduced, which led to a compromised immune response [39]. Gut-associated lymphoid tissue (GALT) is the primary layer of defense against the infectious invasion of the GI. GALT includes lymphoid tissue in the esophagus, stomach, Peyer patches of the small intestine, large intestine, lymphoid follicles, and any lymphocytes in the epithelial layer or lamina propria of the GI [40]. As naïve T cells are often trafficked to the GALT [41], it makes sense that immunosenescence would greatly affect the immune responses arising in the GI.

Given that T cell counts decrease with age, the number of T cells in GALT could also decrease as a result. However, a previous study suggests the opposite: CD4^+^ T cells showed greater recruitment into the GALT of older mice compared with younger mice. Additionally, the recruitment was not correlated with an already-present immune response as (1) cytokines and chemokines were low in the GALT, (2) this T-cell recruitment was unique to GALT as the liver and lungs did not exhibit a similar response, and the (3) increased proliferation of existing CD4^+^ T cells in the GALT was not observed [42]. A study in human samples showed the opposite relationship between T-cell accumulation and aging in GALT, as CD4^+^ T cells decreased with age in the GALT [43]. This difference is interesting because it suggests different mechanisms of GALT immune cell recruitment in mice and in humans. However, the study did not compare the quantity of T cells in the rest of the body compared with the accumulation in the GALT/GI. Therefore, it is still possible that immunosenescence affects the rest of the body more than the GI. Indeed, aging impairs several signaling pathways in the innate immune system, which may result in the manifestation of certain inflammatory conditions and diseases, including diabetes and cancer. 

## 5. Inflammatory Bowel Disease

Inflammatory bowel disease (IBD) describes a group of GI disorders that involves the major inflammation of the GI, with two major subsets: ulcerative colitis (UC) and Crohn’s disease. UC mostly involves inflammation and ulcer formation in the lining of the large intestine and rectum. Crohn’s disease involves chronic inflammation anywhere in the GI, including the esophagus and anus, but usually in the small intestine [44]. One study showed that the prevalence of IBD increased steadily over the past 10 years and was found to be higher in adults than children [45]. With the aging of the population, it becomes pertinent to understand any relationships between senescence/aging and IBD as this knowledge could help with therapies.

IBD is often marked by a dysregulated immune response involving T cells and B cells. One study showed UC-affected mucosa to have higher proportions of B cells than T cells [46], and another study showed naïve B cells to be significantly increased in patients with UC-inflamed colons [47]. Thus, it could be interpreted that increased B-cell proliferation in the large intestine is a biomarker for UC. A subset of B cells termed age-associated B cells (ABCs) are associated with not only aging but also autoimmune diseases, including Crohn’s disease and UC [48]. Several mouse models involving chemically induced colitis have shown excessive or dysregulated TLR signaling [49,50]. This coincides with how TLR signaling primes B cells for ABC transformation [51]. Thus, whereas immunosenescence decreases the immune response in the rest of the body, the increase in ABCs with aging may correlate with an increased risk of IBD. 

Interestingly, TLR signaling was found to be significantly increased in benign polyps but was significantly decreased in polyps taken from patients who eventually had development of colorectal cancer (CRC) [52]. IBD is often associated with CRC due to the combination of chronic inflammation and ulcerative lesions that increases the risk of oncogenic progression [53,54]. In addition, high accumulations of senescent CD8^+^ T cells were found to be associated with greater oncogenic risk [55]. This implies that increased immunosenescence is either a product of or contributes to CRC. Interestingly, inhibition of chronic inflammation in the colon of mice not only increased their life span, but also reduced aging-related changes, especially oxidative DNA damage [56]. Another study showed a similar relationship between UC and cellular senescence: chronic inflammation created an environment that increased DNA damage and telomere shortening, and thereby led to eventual dysplasia and CRC development [57]. 

Further evidence in that IBD is connected to increased cellular senescence came from a study showing that UC increased macrophage counts (which in turn induced senescence), and Crohn’s disease also showed high levels of macrophages and senescence (yet macrophages were not found to be correlated with senescence) [58]. In the same study, macrophages increased senescence via nitric oxide production [58]. The presence of lesions in UC most likely led to the increase in senescence via macrophages, which produce inducible nitric oxide synthase [59]. Another study showed possible therapeutic avenues by manipulation of the telomere length [60]. Whereas telomere shortening was shown to be actively involved in UC development, telomere activators also reduced the expression of common senescence pathways such as p53, and also improved the crypt structure [60]. Recently, rare cases were reported of carcinoma formation in the GALT in the colons of patients with UC [61]. Although GALT is relatively scarce in the colon, these areas still carry a risk of oncogenic development, which requires further research [61].

## 6. Esophagitis

Esophagitis is an inflammation of the esophagus caused by eosinophilia, gastric reflux, or infection. A recent study showed a high prevalence of senescence and cancer-related somatic mutations, including p53 mutations, in the esophagus in older donor tissue [62]. This coincides with the results of a transcriptomic analysis of esophageal epithelium showing mitochondrial dysfunction in the esophagus of older mice [63]. Given the known relationship between p53 and mitochondria, the higher prevalence of p53 mutations may be connected to the dysfunction of mitochondria in older esophageal tissue. Reflux esophagitis has been shown to not only be caused by cytokine inflammation, but also a risk factor for Barrett’s esophagus metaplasia, a precursor to esophageal adenocarcinoma [64]. p53 mutations would most likely lead to increased senescence in the esophagus in older persons, and the added SASP would no doubt further increase cytokine inflammation in the esophagus. Thus, cellular senescence may contribute to a higher risk of esophagitis and eventual oncogenic development.

## 7. *Helicobacter pylori*, Senescence, and Gastric Cancer

**Figure 2:**
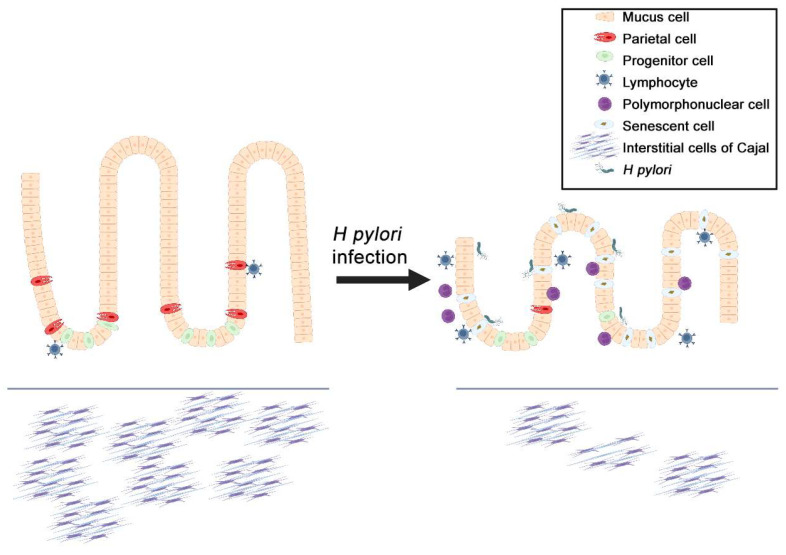
The effect of *Helicobacter pylori* infection and senescent cells on the mucosa of the stomach. *H. pylori* infection leads to oxidative stress on gastric mucosa and the creation of more senescent cells due to DNA damage. This, in turn, leads to the over-secretion of senescence-associated secretory phenotype (SASP) proteins, which creates a highly inflammatory microenvironment in the gastrointestinal tract. Although SASP is usually tumor suppressive due to an immune response, the combination of gastritis and inflammatory factors is tumor promotive, thereby increasing the chances of gastric oncogenesis.

*Helicobacter pylori* is known to infect human stomachs, and *H. pylori* infection remains the major cause of gastric cancer, which involves multiple steps including gastritis, atrophic gastritis, intestinal metaplasia, dysplasia, and gastric cancer development [65,66]. The initial response of the innate immune system to *H pylori* involves gastric epithelial cells. The interaction between *H pylori* and gastric epithelial cells dysregulates signaling pathways leading to the generation of inflammatory and oncogenic processes. These processes include the accumulation of immune cells and DNA damage responses (Figure 2) [67]. As DNA damage directly correlates with the accumulation of senescent cells, *H. pylori* infection may therefore be a key trigger of cellular senescence in the GI. 

*H. pylori* infection-induced atrophic gastritis has been linked to Wnt/β-catenin signaling activation [68,69,70]. Given that aberrant Wnt/β-catenin activation can induce the decrease in gastric ICC numbers with age, the presence of the bacteria may also damage the ICCs in the stomach (Figure 2). In fact, existing evidence shows that ICC numbers are reduced in the presence of *H. pylori* [71]. Given the evidence for correlation of Wnt signaling with the oncogenesis of gastrointestinal stromal tumor (GIST), a common human sarcoma of the GI muscle layer [72], the presence of *H pylori* could lead to higher risks of GIST development [73]. Injuries to the stomach, such as through gastritis, can cause metaplasia of chief cells, and gastric gland cells that release pepsinogen, as well as the loss of parietal cells that are responsible for secreting gastric acid to digest food. Although these changes are designed for wound healing, similar to senescent cells, their presence could be precancerous [74]. Additionally, the pro-inflammatory nature of *H. pylori*-related lesions and nitric oxide may work in tandem with the SASP of the senescent cells to create a highly oncogenic environment [75,76]. Inflammation and SASP increases immune cell recruitment (including polymorphonuclear cells, such as eosinophils and lymphocytes), which correlates with the increased metaplasia in the stomach and contributes to oncogenic progression [77,78]. Together, this suggests that senescence acts synergistically with the microenvironment of the GI to influence the onset of multiple aging-related GI disorders, especially cancer.

## 8. Cellular Senescence in GI Cancers and GIST

The relationship between cellular senescence and cancer is defined by contradiction. One study showed that senescent cells in advanced tumors reduce immune surveillance via SASP, yet cellular senescence was also shown to help tumor-suppressive mechanisms in hepatic carcinoma [79]. Given the senescent cells’ paradoxical roles in tumorigenesis, there is merit in using specific senescence biomarkers to mitigate tumor progression. For instance, the overexpression of tumor suppressors can induce senescence in tumor cells and prevent tumor cell proliferation [80]. The inactivation of genes such as *PTEN* and *MYC* can also cause cellular senescence. *PTEN* loss specifically causes p53 activation, which leads to senescent cell cycle arrest [81]. Thus, GIST may be assumed to have a similar relationship to senescence. However, there is no evidence that GIST can be treated with reinduction of senescence. Given that GIST is believed to develop from ICCs, which do not display senescent phenotypes, it is still possible that a different mechanism is critical for GIST development [34,82].

Instead of cellular senescence, cellular quiescence seems to be more relevant to drug-resistant GIST. Cellular quiescence is a reversible dormancy state of a cell that often leads to a permanent state of senescence [83]. Cellular quiescence is one reason total remission of GIST is infrequent; thus, quiescence proteins are considered as therapeutic targets. One possible example is combining imatinib mesylate, a tyrosine kinase inhibitor of the KIT receptor and the mainstay of treatment for GIST, with the quiescence therapeutic target, the DREAM complex [84], to induce complete cell death. Often, imatinib causes apoptosis in GIST but also results in quiescent cells. As a result, GIST recurrence is highly probable despite imatinib treatment [84]. However, the DREAM complex can be targeted to prevent quiescence via knockdown [85]. Notably, the protein that causes DREAM complex formation, DYRK1A, has an inverse relationship with both cyclin D1 and p21 [86]. The presence of quiescent cells often leads to imatinib-resistant GIST [87]. Furthermore, a recent study showed that imatinib-resistant GIST is highly affected by cyclin D1 targeting via proteasome inhibitors, which often leads to cell cycle arrest as well as the expression of p53 and p21 [88]. Additionally, increased p21 (as well as p53) expression has been associated with a poor prognosis of GIST [89], and p53 is a significant biomarker for GIST progression and proliferation [90,91]. Although these are senescence biomarkers, this is not a clear connection between senescence and GIST.

Evidence does exist, however, for manipulating senescent cells to prevent a tumor-progressive environment in the GI. A recent study showed that the synergism between cellular senescence and *H. pylori* is dependent on CXCR2 signaling [92]. *H. pylori* upregulates CXCR2, which exhibits a positive feedback on p53, and then increases cellular senescence. It is possible that CXCR2 can be pharmaceutically manipulated to decrease senescence caused by *H. pylori* and thus reduce the inflammatory cascade caused by the bacterial infection, subsequently reducing the likelihood of cancer progression [92,93]. 

Similarly, the CXCR4 signaling pathway is involved in tumor invasion and metastasis in CRC [94], the estimated third-leading cause of cancer death [95]. However, this pathway is most important for showcasing the relationship between the senescent tumor cells and immune cell impairment. Senescent tumor cells induce CXCR4 loss in T cells, which leads to impaired leukocyte migration and the inhibition of CD8^+^ T-cell infiltration [96]. This suggests that senescence is integral to CRC development due to the weakened immune system allowing further tumor cell invasion. Additional links between CRC and senescence include the presence of many senescent cells in the stroma of colorectal tumors and the relationship between the SASP and CRC growth [4,97]. Senescent fibroblasts in colonic stroma can enhance adenoma and CRC formation via a paracrine-mediated process that involves GDF15, a member of the TGFβ superfamily [4]. *Escherichia coli* can cause CRC via SASP protein secretion. This bacterial infection can upregulate miR-20a-5p in the colonic mucosa, which negatively regulates SENP1 (a negative regulator of p53) and leads to increased SASP protein expression and thus CRC growth [97]. These CRC studies show that CXCR4, GDF15, and miR-20a-5p are all potential biomarkers that could be targeted to reduce the oncogenic effect of senescent cells on the colon.

## 9. Senotherapeutics and Clinical Trials

Besides manipulating senescence, the destruction of senescent cells may provide optimal therapies. Senolytics are drugs that eliminate senescent cells through apoptosis, and senostatics are drugs that inhibit senescent cells’ function, and together are termed senotherapies [98]. Several clinical trials have already shown the effect of senotherapies on diseases. Senotherapies have been shown to decrease senescent cells in patients with diabetic kidney disease [99]. Circulating levels of α-Klotho, an antiaging protein that can alleviate aging processes, was restored through oral senolytics [100]. Another clinical trial showed senolytic drugs to mitigate the effects of diabetes in obese patients [101]. A study of premalignant pancreatic intraepithelial neoplasia lesions showed that senolytic therapies can reduce the progression of inflammatory lesions into cancer via senescent cell removal [102]. Given that this study showed a similar relationship between pancreatic lesions and senescence as in another study on *H. pylori*-induced atrophic gastritis [92], senolytics could therefore have a similar effect on precancerous gastritis. 

Senotherapies could potentially be used to prevent the tumor-progressive microenvironment in an elderly GI. Unfortunately, senolytic clinical trials regarding the GI are currently scarce. However, a recent study showed that senotherapies can improve intestinal inflammation and overall health in older mice, and another study showed the antiaging potential of destroying senescent cells [103,104]. Thus, there is potential for more variation in senotherapy clinical trials involving the GI in the future.

Increasing evidence has shown that the gasotransmitter hydrogen sulfide (H_2_S) is integral to healthspan-extending interventions, and these effects of H_2_S might be related to a reduced cellular senescence in several tissues [105,106,107]. H_2_S plays this role via the post-translational modification of reactive cysteine residues by protein persulfidation (S-sulfhydration) [108]. The use of exogenous H_2_S might be an appealing application for aging and age-associated diseases but remains challenging due to the short half-life of H_2_S gas in vivo, and the risks of toxicity at high concentrations [109].

## 10. Conclusions

Overall, the evidence suggests that senescence is highly related to the onset of GI diseases and disorders, including cancers. Whether through the disruption of either tumor suppressor genes in the senescence pathway, or the combination of factors resulting from SASP and the GI microenvironment, targeting senescence can influence the development of tumors. As a result, further experimentation in GI cancers and cellular senescence is pertinent, as it would expand our information on the relationship between aging and tumor development. Knowledge of this relationship can then be used to create novel senotherapies that would help reduce the risk of GI oncogenesis in the aging population.

## Data Availability

Not applicable.

## Abbreviations

ABCs	Age-associated B cells
CRC	Colorectal cancer
GALT	Gut-associated lymphoid tissue
GI	Gastrointestinal tract
GIST	Gastrointestinal stromal tumor
H_2_S	Hydrogen sulfide
IBD	Inflammatory bowel disease
ICCs	Interstitial cells of Cajal
IL	Interleukin
SASP	Senescence-associated secretory phenotype
UC	Ulcerative colitis
**Gene/Protein Expansions**	
CXCR	C-X-C motif chemokine receptor
DREAM	Dimerization partner, RB-like, E2F and multi-vulval class B
DYRK1A	Dual specificity tyrosine-phosphorylation-regulated kinase 1A
ERK	Extracellular signal-regulated kinase
GDF15	Growth differentiation factor 15
KIT	KIT proto-oncogene, receptor tyrosine kinase
miR-20a-5p	MicroRNA-20a-5p
MYC	MYC proto-oncogene, bHLH transcription factor
PTEN	Phosphatase and tension homolog
PUMA	p53 upregulated modulator of apoptosis
SENP1	SUMO specific peptidase 1
TGFβ	Transforming growth factor β
TLR	Toll-like receptor
Wnt	Wingless/integrated
